# Large Area High‐Performance Thin Film Solid Oxide Fuel Cell with Nanoscale Anode Functional Layer by Scalable Reactive Sputtering

**DOI:** 10.1002/advs.202502504

**Published:** 2025-05-29

**Authors:** Kyoungjae Ju, Seongkook Oh, Jong Hyuk Lee, Hyong June Kim, Hyunmin Kim, Sung Eun Jo, Juhwan Lee, Byung Chan Yang, Jisung Yoon, Dong Won Shin, Wanwoo Park, Ji‐Won Son, Young‐Beom Kim, Sungeun Yang, Jihwan An

**Affiliations:** ^1^ Department of Mechanical Engineering Pohang University of Science and Technology (POSTECH) Pohang 37673 Republic of Korea; ^2^ Energy Material Research Center Korea Institute of Science and Technology (KIST) Seoul 02792 Republic of Korea; ^3^ Division of Nanoscience and Technology KIST School, Korea University of Science and Technology (UST) Seoul 02792 Republic of Korea; ^4^ Department of Mechanical Engineering Hanyang University Seoul 04763 Republic of Korea; ^5^ Research and Development Team AVACO Co., Ltd Daegu 42724 Republic of Korea; ^6^ Graduate School of Energy and Environment (KU‐KIST Green School) Korea University Seoul 02841 Republic of Korea

**Keywords:** nanostructured anode functional layer, reactive sputtering, solid oxide fuel cells, thin‐film solid oxide cells, triple‐phase boundary

## Abstract

For high‐performance thin‐film solid oxide cells (TF‐SOCs), a nanostructured anode functional layer (n‐AFL) that can prolong the triple‐phase boundary (TPB) is crucial, particularly for low‐temperature operation. However, the implementation of n‐AFL (usually >1 µm in thickness) has critical issues in scale‐up and productivity. Here, the study successfully demonstrates a large‐area, high‐performance TF‐SOFC with an n‐AFL fabricated via mass‐production‐compatible reactive magnetron sputtering. The cell with optimized n‐AFL by adjusting crucial reactive‐sputtering process parameters, i.e., oxygen partial pressure and sputtering power, shows superior performance compared to that of the cell without n‐AFL: the reduction both in ohmic and anodic polarization resistances by 63% and 34%, respectively, and the improvement in maximum power density by 89% (0.705 W cm^−2^ vs 1.333 W cm^−2^) at 650 °C. When employed in large‐scale cell (4 × 4 cm^2^), the TF‐SOFC with n‐AFL showed 19.4 W at 650 °C.

## Graphical abstract

The high‐power density (1.33 W cm^−2^ at 650 °C) thin film solid oxide fuel cells (TF‐SOFCs) with nanoscale anode functional layer (n‐AFL) by high‐speed (>2 µm h^−1^) reactive magnetron sputtering process is successfully demonstrated. The large‐area (16 cm^2^) TF‐SOFCs with n‐AFL exhibited a power output of 19.36 W at 650 °C, which is the highest performance for TF‐SOFCs fabricated via reactive sputtering.

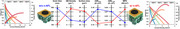

## 1 Introduction

Solid oxide fuel cells (SOFCs) are promising energy‐conversion devices that can directly convert the chemical energy of fuels into electricity with high efficiency, sustainability, and scalability.^[^ ^1^, ^2^ ^]^ However, the high operating temperature of SOFCs (>800 °C) causes challenges, such as long start‐up time, long‐term stability, and material compatibility issues. Therefore, research focused on developing intermediate‐ and low‐temperature SOFCs operating between 450–650 °C.^[^ ^3^, ^4^ ^]^ Nevertheless, lowering the operating temperature causes an undesirable increase in ohmic and polarization losses due to slower ionic conduction and reaction kinetics at the electrolyte and electrode, respectively. Therefore, it is important to reduce the thickness of the electrolyte and promote reactions at the electrodes to mitigate these issues.

Thin‐film SOFCs (TF‐SOFCs), which have thin film electrolytes (<1 µm) could be favorable candidates for low‐temperature SOFCs. The thin‐film electrolyte mitigated the increased resistance caused by the decreased temperature and oxygen‐ion conductivity in the electrolyte.^[^ ^5^, ^6^, ^7^ ^]^ Nevertheless, the enhancement of the electrochemical performance at the electrode is still necessary to achieve high electrochemical performance in TF‐SOFC. Furthermore, the mechanical stability of the TF‐SOFC is largely limited by the fragile nature of thin film components, especially at elevated temperature. Therefore, the nanoscale anode functional layer (n‐AFL) between the anode and electrolyte of an anode‐supported cell, usually in a NiO‐doped zirconia/ceria cermet, was proposed. The n‐AFL extends the triple‐phase boundary (TPB) length and therefore improves the anode kinetics as well as the overall cell performance, while significantly substantiating the mechanical stability of the thin‐film electrolyte by linking the scale gap between the microporous anode support and the nanotight thin‐film electrolyte.^[^ ^8^, ^9^, ^10^ ^]^

Various thin film deposition methods were explored to fabricate n‐AFL for TF‐SOFCs, namely, pulsed laser deposition (PLD) and radio frequency (RF) magnetron sputtering.^[^ ^8^, ^9^, ^10^, ^11^, ^12^, ^13^, ^14^ ^]^ The Ni agglomeration in PLD n‐AFL was successfully suppressed by optimizing the post‐heat treatment, which was later proven to be more than an order of magnitude higher than that of the TPB density compared to that of the conventional anode by 3D topographical analysis.^[^ ^10^, ^14^ ^]^ The n‐AFLs fabricated by PLD and RF sputtering effectively link the anode support and thin electrolyte and enhance the structural integrity and thermostability of multiscale TF‐SOFC.^[^ ^10^, ^11^, ^12^, ^13^, ^15^, ^16^, ^17^ ^]^ Even though the PLD and RF sputtering processes have successfully fabricated n‐AFL for TF‐SOFCs, they still show limitations: the PLD process can only deposit a limited area. RF sputtering process allows for large‐area deposition, although the deposition rate is usually limited to <100 nm h^−1^, which is not practically useful considering the effective thickness of n‐AFLs (usually ≥ 2 µm).^[^ ^5^, ^18^, ^19^, ^20^ ^]^

In this regard, reactive sputtering can provide a viable large‐area, high‐speed deposition tool for thin‐film components, for example, electrolytes and n‐AFLs, for TF‐SOFCs. In reactive sputtering, the metal atoms ablated from the metal target react with the reactive gas plasma (e.g., O_2_, N_2_ plasma) so that ceramic films such as oxides and nitrides could be deposited. Due to the high sputter yield of metal targets, the deposition rate is usually even higher than that of RF sputtering, which uses a ceramic target (e.g., RF sputtering ≈200 nm h^−1[^ ^21^ ^]^ versus reactive sputtering >2 µm h^−1^ (in this work) for Y:ZrO2 deposition); therefore, it has become a valuable commercial process for depositing dielectrics, semiconductors, etc. Nevertheless, the properties of films can vary widely from metals to semiconductors and insulators, depending on the extent of the reaction; therefore, precise control of the process parameters is extremely important for depositing films with a target composition. Therefore, limited number of previous publications exist on the successful employment of reactive‐sputtered components such as n‐AFL, which is usually composed of multiple elements, in high‐performance TF‐SOFCs.^[^ ^22^, ^23^, ^24^ ^]^

**Figure 1:**
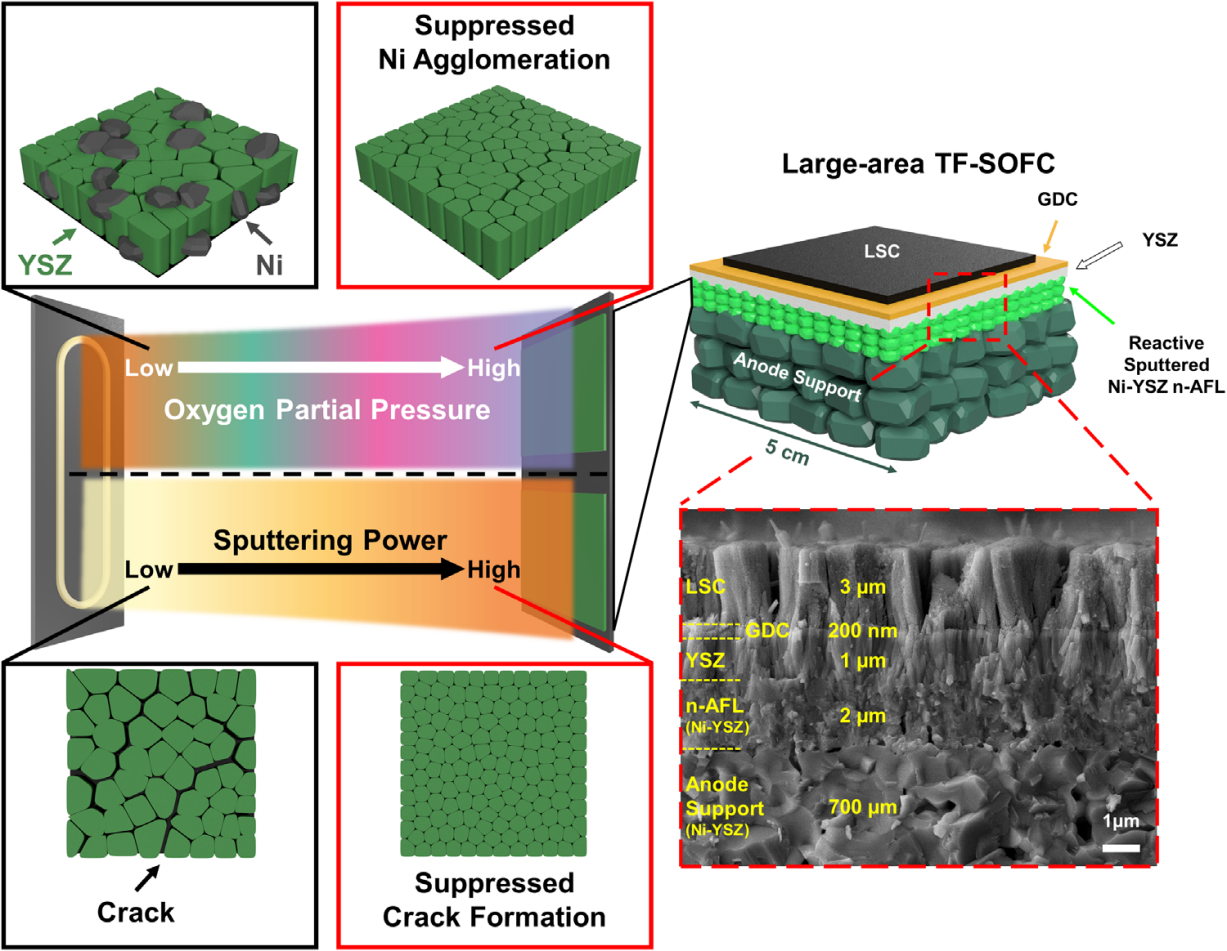
Schematic diagrams of n‐AFL fabrication process using reactive magnetron sputtering by adjusting parameters: oxygen partial pressure and sputtering power with a cross‐sectional SEM image of TF‐SOFC with the n‐AFL.

Here, we demonstrate a large‐area high‐performance thin film SOFCs with n‐AFL by optimizing the high‐speed (>2 µm h^−1^) reactive magnetron sputtering process. The parametric effects of the oxygen partial pressure and sputtering power on the composition and microstructure of reactive sputtered n‐AFL were investigated, whose properties were further improved by thermal annealing (**Figure** **1**). Ohmic and anodic polarization resistances decreased by 63% and 34%, respectively, and the maximum power density increased by 89%, leading to 1.33 W cm^−2^ at 650 °C, by adopting reactive sputtered n‐AFL. In large‐scale cell (4 × 4 cm^2^), the TF‐SOFC with n‐AFL showed 19.4 W at 650 °C. These results suggest that n‐AFL deposited via reactive magnetron sputtering may contribute to facile industrial applications of high‐performance TF‐SOFCs.

## 2 Results and Discussion

### 2.1 Effect of Process Parameters on Physical and Chemical Properties of n‐AFL

**Figure 2:**
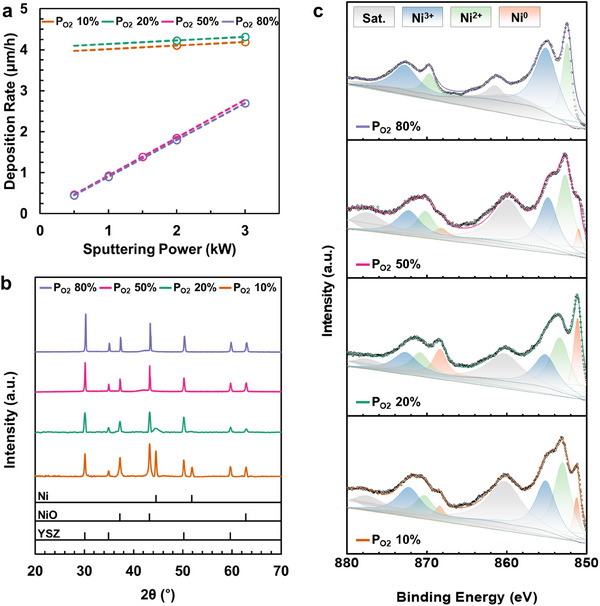
a) Deposition rate depends on the sputtering power and oxygen partial pressure (P_O2_/P_Ar+O2_). b) XRD, and c) XPS spectra (Ni 2p) of reactive sputtered NiO‐YSZ with respect to oxygen partial pressure (sputtering power @2 kW).

The effects of the sputtering power and oxygen partial pressure, which are two important parameters of the reactive sputtering process, on the deposition rate and composition of the sputtered NiO‐yttria‐stabilized zirconia (NiO‐YSZ) n‐AFL are shown in **Figure** **2**. The deposition rate exhibited linear correlation with the sputtering power due to enhanced sputter yield (Figure 2a).^[^ ^25^ ^]^ The deposition rate became saturated when the oxygen partial pressure exceeded 50%, suggesting the oxidation of the metal Ni/Y/Zr target surface by the excessive introduction of the reactive gas (Figure S1, Supporting Information).^[^ ^25^, ^26^ ^]^ Volumetric contents of Ni exhibited similar trend to the deposition rate whereas Zr and Y contents show opposite behavior (Figure S2, Supporting Information). X‐ray diffraction (XRD) analysis (Figure 2b) reveals the crystalline structure of the as‐deposited NiO‐YSZ n‐AFL. All as‐deposited films contained peaks corresponding to metallic Ni (JCPDS card no. 01‐071‐4653), Ni oxide (JCPDS card no. 01‐072‐1464), and YSZ (JCPDS card no. 00‐030‐1468). The intensities of the metallic Ni (111) peak at 2θ = 44.3° and (200) peak near 2θ = 51.6° decrease as the oxygen partial pressure increases. Metallic Ni peaks are not observed when the oxygen partial pressure exceeds 50%. The high‐resolution X‐ray photoelectron spectroscopy (XPS) spectra of the Ni 2p region (850–880 eV, Figure 2c) show that the metallic Ni peaks near 851 eV in the 10%, 20%, and 50% samples disappeared in the 80% sample, indicating the oxidation of metallic Ni to the oxide phase, which also corresponds well with that of the XRD results.

**Figure 3:**
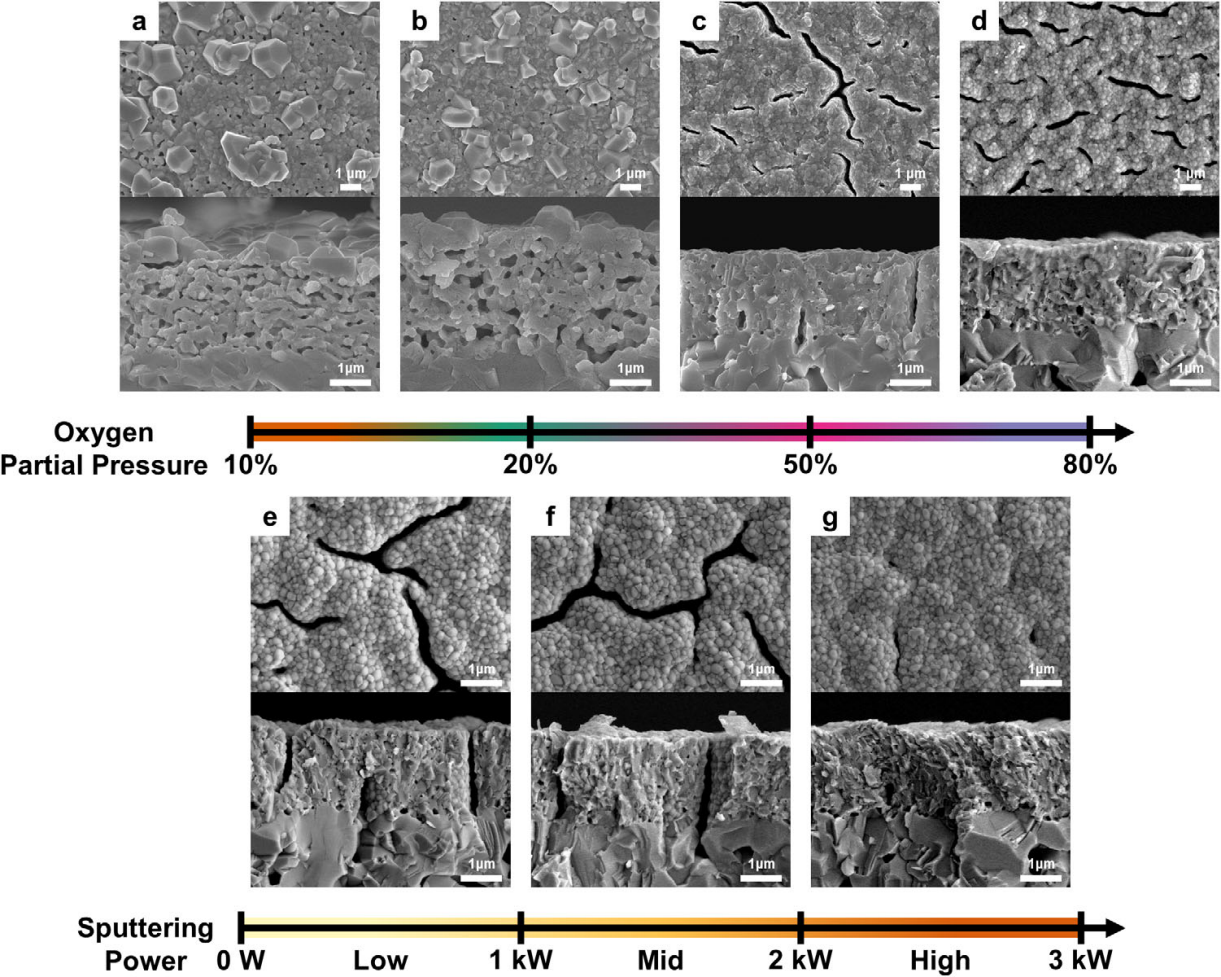
SEM images of 1200 °C‐annealed NiO‐YSZ n‐AFL at different a–d) oxygen partial pressure conditions with constant sputtering power of 2 kW and e‐g) sputtering power conditions with a constant oxygen partial pressure of 80%.

**Figure** **3**a–d and Figure S3 (Supporting Information) illustrate the morphological changes of reactively sputtered NiO‐YSZ n‐AFL subjected to different oxygen partial pressure (P_O2_/P_Ar+O2_) conditions after undergoing a 1 h annealing at 1200 °C. In the top view, the as‐deposited low P_O2_ (≤ 20%) n‐AFL samples show 29% bigger grains (≈803 nm) than the high P_O2_ (≥ 50%) samples (≈622 nm), which implies the dense formation of electrochemically active triple phase boundaries in the high P_O2_ samples (Figures S3 and S4, Supporting Information). The cross‐sectional view of the as‐deposited films displayed tapered columnar grains, which became more densely packed with clear facets as P_O2_ increases (Figure S3, Supporting Information). After the thermal treatment at 1200 °C, the low P_O2_ n‐AFLs exhibited a prominent structural change; the aggregated Ni grains emerged on the surface, causing the significant volume loss in bulk n‐AFL. Contrarily, the high P_O2_ (> 50%) n‐AFLs partially showed the crack formation after the annealing, with marginal porosity formation (Figure 3; Figures S5 and S6, Supporting Information).

The effect of varying sputtering power (0.5 to 3 kW), while keeping P_O2_ of 80% as constant on the n‐AFL morphology was also investigated by SEM analysis. While all the as‐deposited n‐AFLs exhibited dense and vertically oriented columnar grains of 470–650 nm (Figures S7 and S8, Supporting Information), clear differences were observed in the pore structures formed after annealing (Figure 3e–g). When the sputtering power was >2 kW, the average porosity of the n‐AFLs after annealing decreased from 3.6% to 0.8% (Figure S9, Supporting Information), which seemed to be due to the facilitated diffusion of the atoms on the surface, leading to building a denser structure by higher energy ion bombardment.^[^ ^27^ ^]^ Therefore, we can conclude that the dense and thermally stable NiO‐YSZ film, which is optimal for n‐AFL, can be optimally fabricated at high oxygen partial pressure (≥50%) and high sputtering power (≥2 kW), due to facilitated oxidation and energetic ion bombardment, respectively.

### 2.2 Electrochemical Characterizations of TF‐SOFCs with n‐AFL

**Figure 4:**
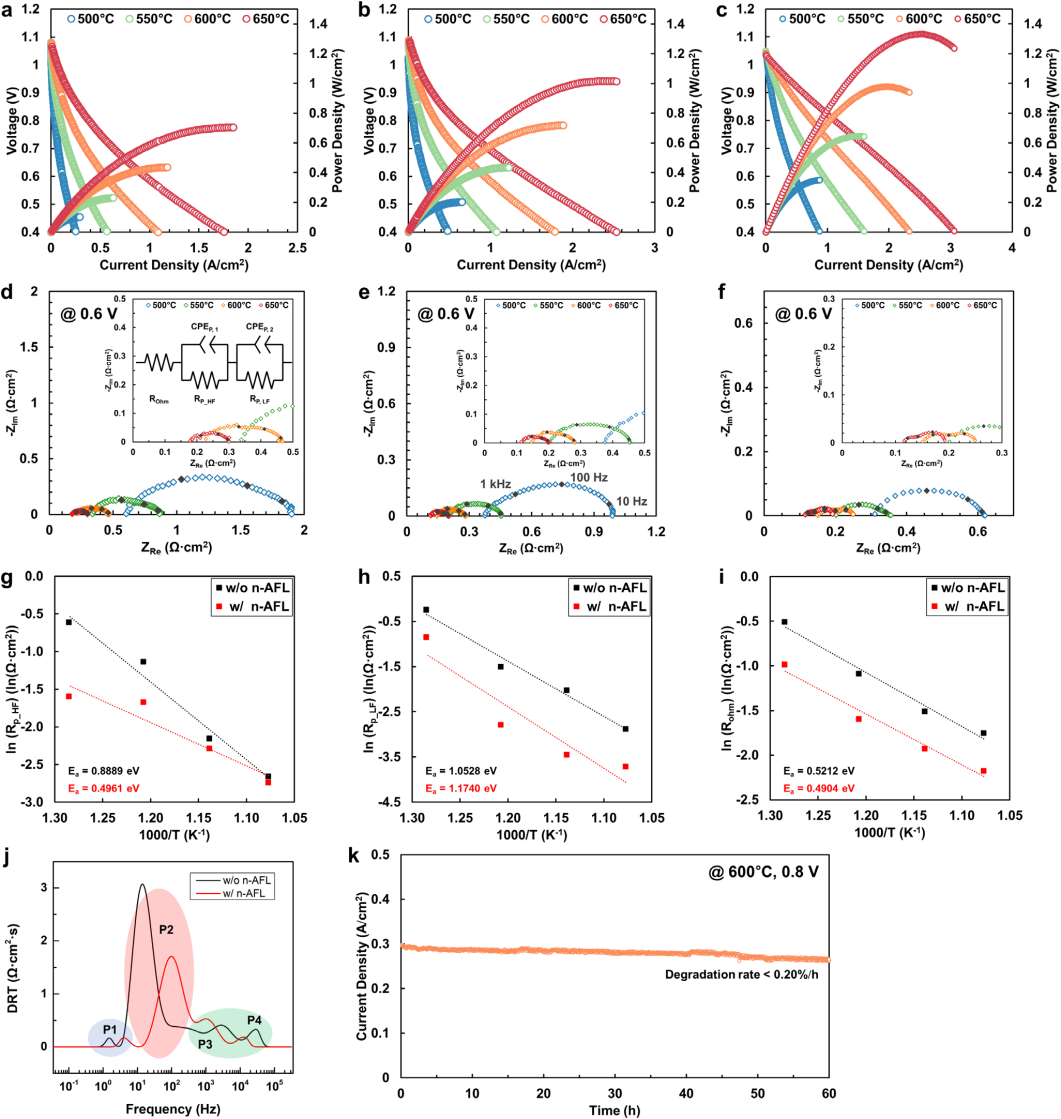
Electrochemical performance of the TF‐SOFCs a) without and b) with the n‐AFL on the polished Ni‐YSZ anode supports, and c) the cell with the n‐AFL on the unpolished anode support. EIS spectra of the cells d) without and e) with n‐AFL on the polished anode supports, and f) the cell with the n‐AFL on the unpolished anode support. Arrhenius plots of polarization resistances of g) high‐frequency and h) low‐frequency region, and i) ohmic resistance (R_P_HF_, R_P_LF_, and R_ohm_ in the equivalent circuit model, respectively) at 0.6 V. j) Distribution of relaxation time (DRT) analysis of the cells with and without n‐AFL. k) Long‐term stability under a constant voltage of 0.8 V at 600 °C.

**Figure** **4** shows the effect of the reactive‐sputtered n‐AFL on the electrochemical performance of the anode‐supported TF‐SOFCs. Foremost, the cells exhibit the open circuit voltages (OCVs) of 1.04–1.09 V, which is close to the thermodynamic voltage (1.08–1.12 V at 500–650 °C) and matches well with those of the earlier reported TF‐SOFCs.^[^ ^5^, ^7^, ^11^, ^12^, ^19^, ^20^, ^28^, ^29^ ^]^ The electrochemical performance of the TF‐SOFCs is illustrated in Figure 4a–c: a) a polished anode‐supported cell without n‐AFL, b) a polished anode‐supported cell with n‐AFL, c) a unpolished anode‐supported cell with n‐AFL. The TF‐SOFC without n‐AFL on the unpolished support did not show a meaningful OCV due to mechanical instability at elevated temperature (Figure S10, Supporting Information). The cell with the reactive‐sputtered n‐AFL at optimal conditions (sputtering power: 3 kW, P_O2_ / P_Ar+O2_: 80%) showed maximum power densities (MPDs) of 1.02, 0.72, 0.44, and 0.20 W cm^−2^ in 650, 600, 550, and 500 °C, respectively, which increase up to 95% in MPDs compared to that of the cell without the n‐AFL. Furthermore, the TF‐SOFCs fabricated on the unpolished Ni‐YSZ anode supports demonstrate even higher MPDs; 1.33, 0.98, 0.65, and 0.35 W cm^−2^ at 650, 600, 550, and 500 °C, respectively, which shows 89% increase in MPD at 650 °C (Figure 4c). The implementation of n‐AFL not only smoothens the substrate top allowing the deposition of dense thin film electrolyte, but also enhances the electrochemical performance due to the increased TPB length as discussed later.

Electrochemical impedance spectroscopy (EIS) was performed for more in‐depth electrochemical analysis of the cells. The EIS spectra were deconvoluted into three parts: Ohmic, high‐, and low‐frequency polarization impedances. The ohmic resistance (>10 kHz) was independent of the cell voltage, whereas the polarization resistance showed substantial variation depending on the applied overpotential (Figure S11, Supporting Information). Furthermore, the polarization impedance can be divided into high‐ (10 k–100 Hz) and low‐frequency (100–1 Hz) regions attributed to the anode and cathode, respectively. The R_ohm_, R_p_HF_, and R_p_LF_ in the equivalent circuit correspond to the ohmic, anode, and cathode resistances, respectively (Figure S12, Supporting Information). Figure 4d,e shows that the n‐AFL reduces the ohmic resistance from 0.60 to 0.37 Ω·cm^2^ at 500 °C, which is a 38% decrease and shows similar trends at all measured temperatures. The anode resistance was reduced from 0.070 to 0.065 Ω·cm^2^, showing only 8% lower value at 650 °C; the amount of decrement was extended by lowering the operating temperature, so the anode resistance was decreased from 0.54 to 0.20 Ω·cm^2^, showing 63% lower value at 500 °C. The EIS spectra of the cell with n‐AFL on the unpolished support exhibited even lower anode resistance of 0.026 Ω·cm^2^ at 650 °C (Figure 4f). The Arrhenius plots of anode, cathode polarization, and ohmic resistances obtained at the cell voltage of 0.6 V are depicted in Figure 4g–i, respectively. The activation energies of the ohmic process with and without n‐AFL samples are similar, with values of 0.49 and 0.52 eV, respectively. Similarly, the cathodic polarization activation energies show close values of 1.05–1.17 eV, respectively. However, the activation energies of anode polarization process with and without n‐AFL samples are 0.50 and 0.89 eV, respectively, and a decrease of 44% by adopting n‐AFL is observed. Figure 4j illustrates the distribution of relaxation times (DRT) analysis derived from the EIS data at 550 °C, identifying four distinct peaks, P1, P2, P3, and P4 (from low to high frequency), corresponding to those of the different electrochemical processes. DRT analysis highlights a prominent reduction in the intensity of the P2 peak (≈10 Hz < *f* < ≈1 kHz), which reflects mixed contributions from the oxygen surface exchange at the cathode and the charge transfer reaction and ionic transport at the AFL.^[^ ^30^, ^31^, ^32^, ^33^ ^]^ Considering that the cathode remained identical between the samples, such a significant reduction in the P2 peak intensity seems to imply an improvement in charge transfer and ionic transport in the n‐AFL. The long‐term stability of the n‐AFL implemented cell is shown in Figure 4k. At a constant voltage of 0.8 V and a temperature of 600 °C, the TF‐SOFC was continuously operated for 60 h. No huge degradation was observed with a degradation rate of <0.2%/h.

**Figure 5:**
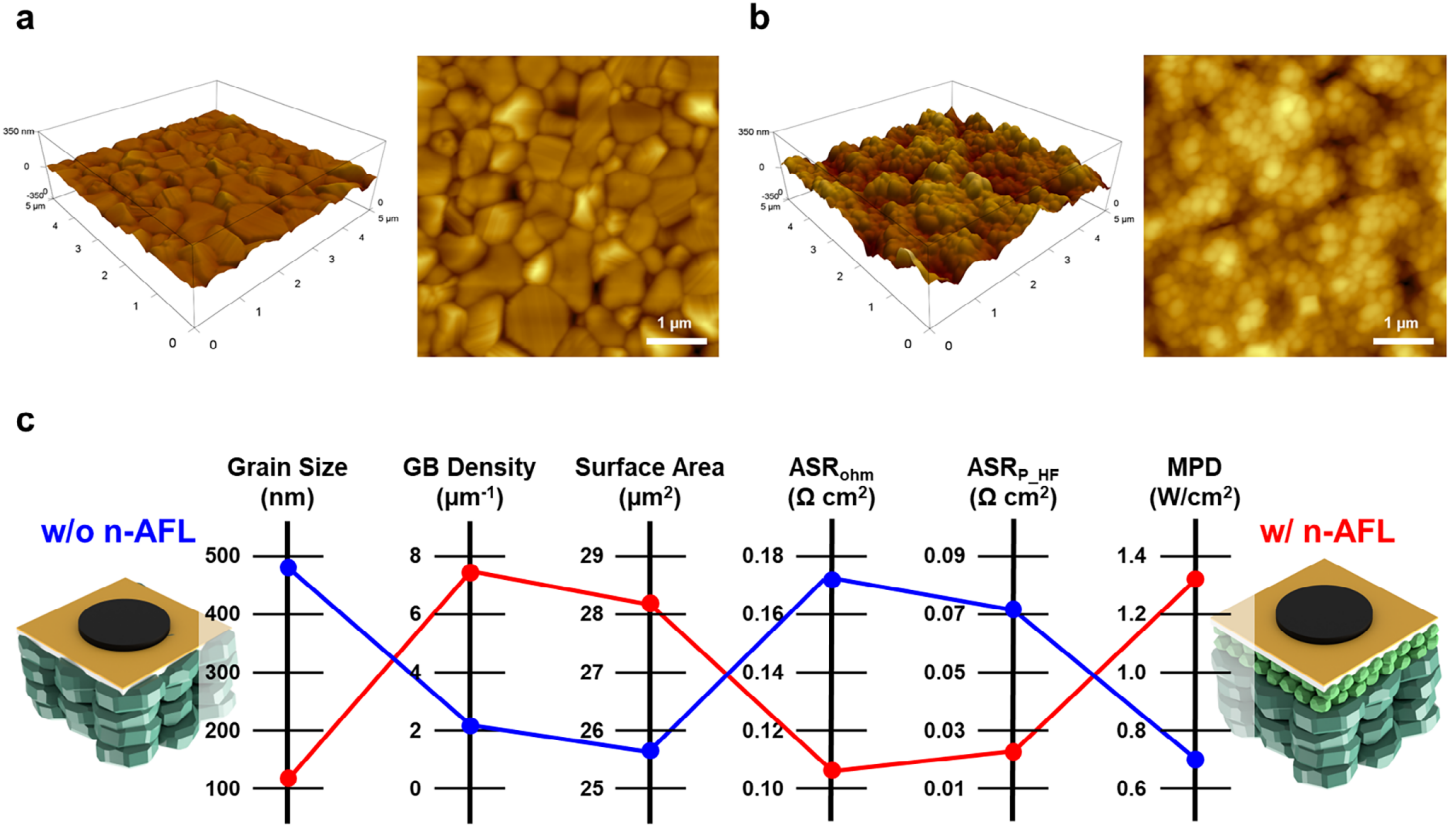
AFM images (5  ×  5 µm^2^) of a) polished anode support, and b) n‐AFL deposited on the unpolished anode support. c) Comparison of the quantified microstructural parameters and electrochemical values measured at 650 °C.

To understand the relationship between the microstructure and electrochemical performance, the interfaces between the anode and electrolyte after annealing at 1200 °C were analyzed by atomic force microscopy (AFM). The AFM test was conducted for polished anode support, (**Figure** **5**a; Figure S13, Supporting Information) unpolished anode support (Figure S14, Supporting Information) and n‐AFL implemented unpolished anode support (Figure 5b; Figure S15, Supporting Information). The average diameter of grains on n‐AFL surface (132 nm) was 70% smaller than that on NiO‐YSZ anode support surface without n‐AFL (470 nm) (Figure 5a,b). The density of surface grain boundaries (GB), known to show faster kinetics and higher electrocatalytic activity, become more than doubled (Figure 5c; Figure S16, Supporting Information). The n‐AFL (28.1 µm^2^) also demonstrates 9.3% larger surface area compared to that of the Ni‐YSZ anode support surface without n‐AFL (25.7 µm^2^).

Consequently, the reduction in resistance observed in the EIS analysis by employing n‐AFL may have originated from the microstructural characteristics of the n‐AFL, which may be due to the increased surface area leading to a more intimate interface between the anode and electrolyte, resulting in a lower interfacial contact resistance. Furthermore, the reduction in the anode polarization resistance and activation energy seems to be due to the larger surface GB density, stemming from the nanoscale fine grains in n‐AFL, which turns into the high‐density TPBs after reduction. This combination of improved ohmic and polarization processes contributed to the superior electrochemical performance of the TF‐SOFC with the reactive‐sputtered n‐AFL.^[^ ^10^, ^34^, ^35^ ^]^

**Figure 6:**
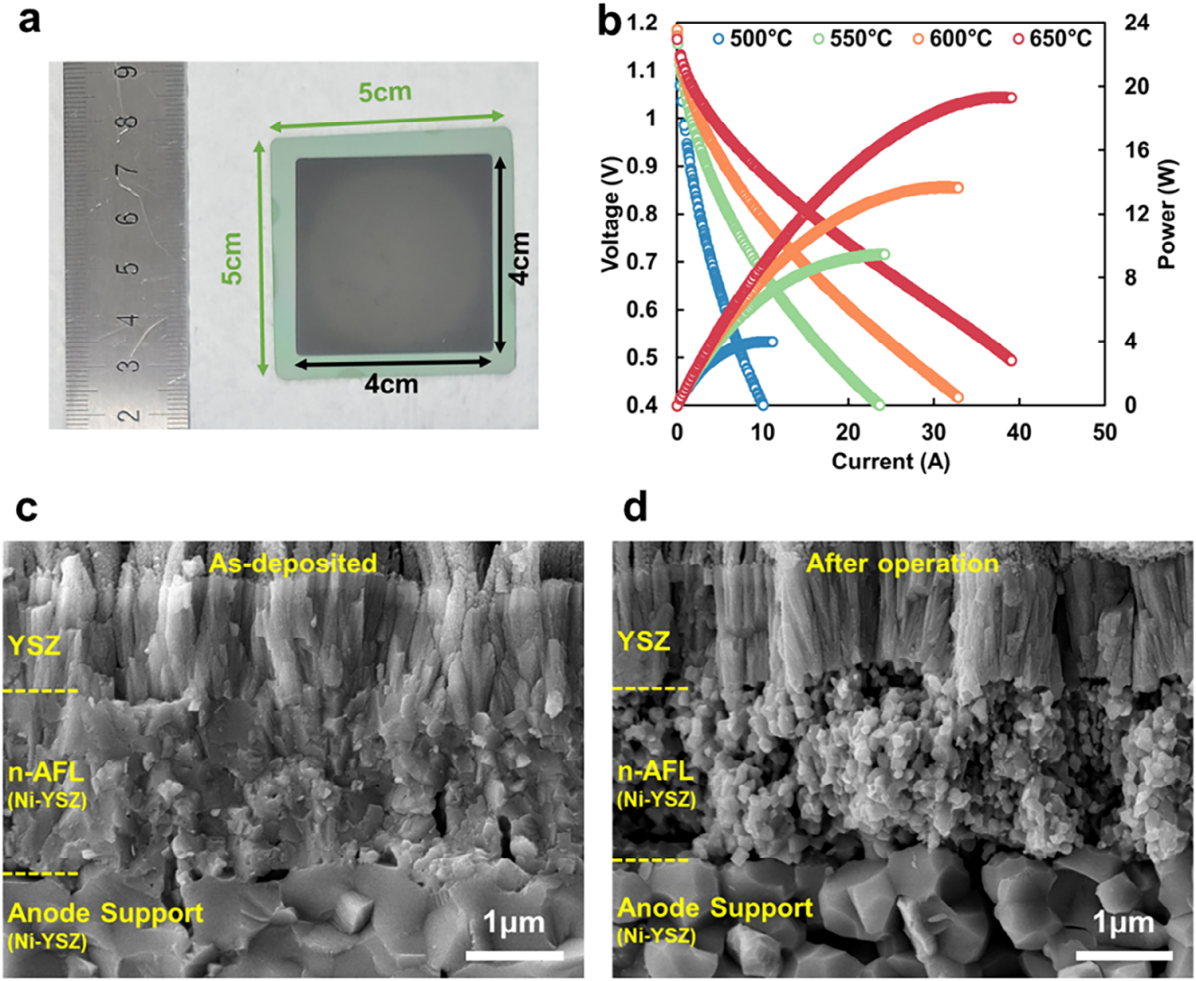
a) Image of the as‐fabricated large‐area TF‐SOFC with n‐AFL with the active area of 16 cm^2^. b) I‐V‐P curve of n‐AFL implemented large‐area TF‐SOFC. SEM images of c) as‐deposited and d) after‐operated n‐AFL.

The key issues in the commercialization of TF‐SOFC technology include scalable fabrication and production speed while maintaining a promising electrochemical power output. After confirming the high‐speed fabrication (>2 µm h^−1^) of n‐AFL for high‐performance TF‐SOFCs, we further demonstrated >10 W‐scale large‐area TF‐SOFC by implementing the mass‐production compatible reactive magnetron sputtering to fabricate n‐AFL on the unpolished anode support (**Figure** **6**a). The OCVs of a large‐area cell with an active area of 16 cm^2^ are 1.15–1.17 V at 650–500 °C, which corresponds well to the OCV of button cells (1.05 V), confirming the gas tightness throughout the large area cell. Additionally, the maximum power output values of the large‐area TF‐SOFC are 19.36, 13.74, 9.49, and 4.01 W at 650, 600, 550, and 500 °C, respectively (Figure 6b). These values correspond to maximum power densities of 1.21, 0.86, 0.59, 0.25 W cm^−2^. The slightly lower power density in the large area cell compared to the button cell could be attributed to factors such as current collection, local variations in temperature, and/or pressure. The microstructures of the interface between the n‐AFL and the YSZ electrolyte were further investigated via cross‐sectional SEM images before (Figure 6c) and after operation (Figure 6d), wherein n‐AFL acted as a uniform and robust support for maintaining the cell structure even at a large scale (Figure S17, Supporting Information).

**Figure 7:**
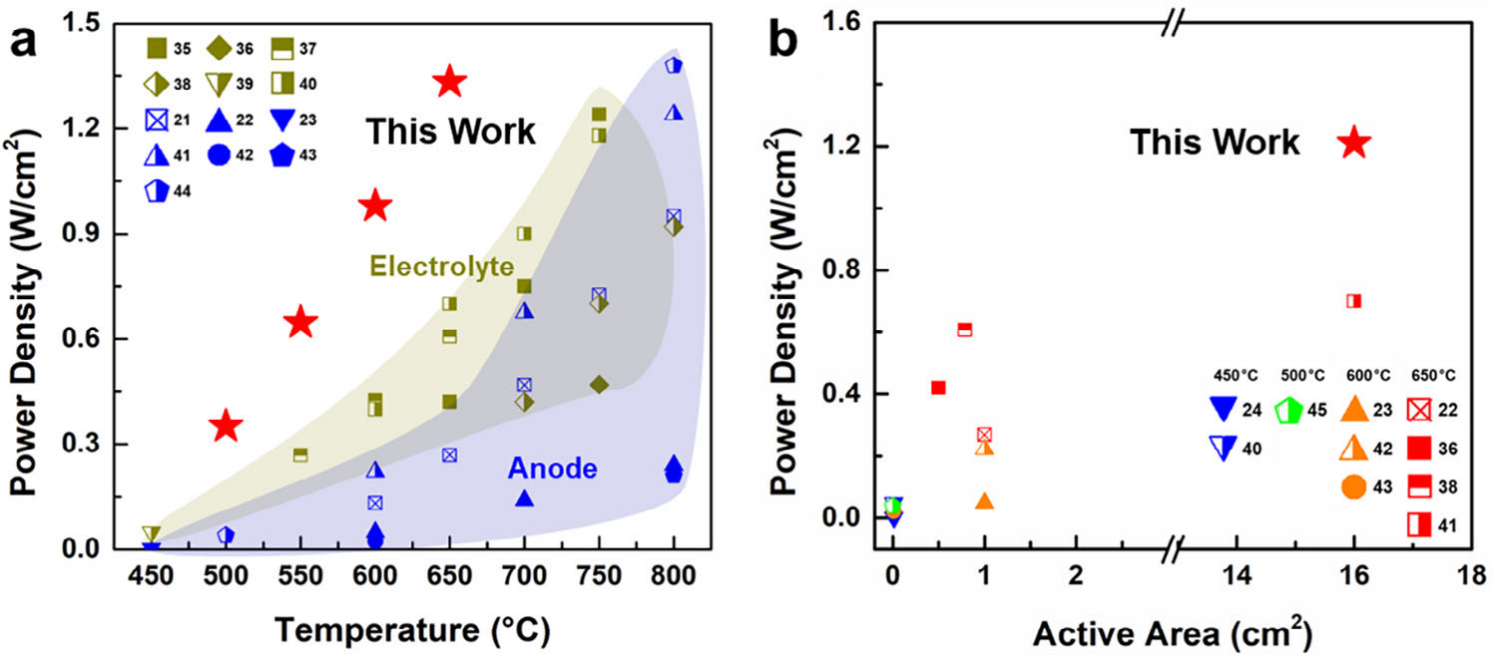
Comparison of power density in this work with that of earlier reported SOFCs using a reactive sputtering method with respect to a) the operating temperature and b) the active area operated below 650 °C.

To the best of our knowledge, the TF‐SOFC with the optimized reactive‐sputtered n‐AFL in this study demonstrated superior electrochemical performance compared to that of earlier reports utilizing the reactive sputtering method to fabricate electrolytes (gold area),^[^ ^36^, ^37^, ^38^, ^39^, ^40^, ^41^ ^]^ and anode/AFL (blue area) (**Figure** **7**a).^[^ ^22^, ^23^, ^42^, ^43^, ^44^, ^45^ ^]^ Furthermore, the cell with n‐AFL displays the highest cell performance with the largest active area among the reported studies on intermediate‐ to low‐temperature (<650 °C) SOFCs using reactive sputtering (Figure 7b). Moreover, when tested in electrolysis mode, the cell with reactive sputtered n‐AFL exhibited a current density of 0.7 A cm^−^^2^ at thermal neutral voltage (1.3 V) at 650 °C, confirming the structural versatility of n‐AFL for both electrolysis and fuel cell applications (Figure S18, Supporting Information). More in‐depth investigations in this area are currently ongoing in our laboratory.

## 3 Conclusion

Here, we present the development of an n‐AFL for TF‐SOFCs using reactive magnetron sputtering. Reactive sputtering with a Ni/Zr/Y metal target allowed control of the chemical composition and achieved a high deposition rate of ≈2.7 µm h^−1^. The n‐AFL deposited by reactive sputtering exhibits desirable characteristics of dense and vertically oriented small columnar grains, as well as maintaining low porosity after the annealing at 1200 °C. The fabrication of the n‐AFL was optimized by adjusting two parameters of reactive sputtering, which are oxygen partial pressure and sputtering power. The implementation of optimized n‐AFL to the TF‐SOFCs led to a promising electrochemical performance of 1.33 W cm^−2^ at 650 °C. Furthermore, we demonstrate the scalability of the n‐AFL process by fabricating large‐area TF‐SOFCs with an active area of 4  ×  4 cm^2^. To the best of our knowledge, the large‐area TF‐SOFCs with n‐AFL exhibited a total power output of 19.36 W at 650 °C, which is the highest electrochemical performance reported for TF‐SOFCs fabricated via reactive magnetron sputtering. We believe that the demonstration of a high‐performance TF‐SOFC with a high‐throughput and scalable fabrication process shown in this study has significant implications for the commercialization of a broad range of thin‐film‐based energy devices such as solid oxide fuel cells, electrolysis cells, and solid‐state batteries.

## 4 Experimental Section

### Reactive Sputtering of n‐AFL

The 2 µm thick nickel oxide‐yttria stabilized zirconia (NiO‐YSZ) n‐AFL was deposited on a 5 × 5 cm^2^ powder‐based NiO‐YSZ (60:40 wt%) anode support substrate (Kceracell, Korea) using reactive sputtering. Reactive sputtering was performed using an in‐line magnetron sputtering system, ZV 6000 (AVACO Co. Ltd., Korea), with a 486 × 86 mm^2^ Ni/Zr/Y metal alloy sputtering target (Advantec Korea Co. Ltd., Korea) in a mixed atmosphere of Ar and O_2_. The partial pressure of oxygen was determined by dividing the flow rate of the reactive gas by the overall flow rate. The working pressure was maintained at 3 mTorr throughout the sputtering process, and the oxygen partial pressure (P_O2_/(P_O2_+P_Ar_)) varied from 10%–80%. The sputtering power was adjusted from 0.5 to 3 kW, categorized as “Low” (< 1 kW), “Mid” (1–2 kW), and “High” (3 kW). The deposition was performed at room temperature. Finally, the 1 h thermal treatment at 1200 °C was followed to strengthen the YSZ network and suppress the Ni agglomeration after the n‐AFL fabrication by reactive sputtering.^[^ ^14^ ^]^

### Full Cell Preparation

For the full‐cell fabrication, anode supports were prepared with and without surface polishing. After n‐AFL fabrication on the anode supports, a thin‐film YSZ electrolyte, gadolinia‐doped ceria (GDC) diffusion barrier layer, and lanthanum strontium cobalt oxide (La_0.6_Sr_0.4_CoO_3_, LSC) cathodes were fabricated. The electrolyte was fabricated using reactive magnetron sputtering (ZV 6000, AVACO Co. Ltd., Korea) at room temperature. The oxygen partial pressure, working pressure, and sputtering power were maintained 50%, 3 mTorr, and 500 W, respectively. The GDC buffer layer was deposited at 700 °C, under 5 mTorr of Ar ambient pressure, using 100 W of radio frequency power with a 2‐inch diameter target. The LSC cathodes were deposited with the PLD system, which use KrF excimer laser source (λ = 248 nm, COMPEX Pro 201F, Coherent, USA). The LSC target was prepared using La_0.6_Sr_0.4_CoO_3₋δ_ powder (Kceracell, Republic of Korea). The powder was uniaxially pressed into a 3 cm‐diameter pellet under a load of 600 kg, followed by sintering at 1300 °C for 3 h. The deposition temperature was maintained at room temperature, and the deposition pressure was 100 mTorr O2. The distance between the target and substrate was kept as 6.5 cm, and the laser fluence was 2.5 J cm^−2^ on the LSC target. The target thickness of LSC cathode layer was 3 µm. To enhance film deposition adhesion and crystallinity, the cell was sintered at 650 °C for 3 h.

### Physical and Chemical Characterization

The top and cross‐sectional morphologies of the deposited films were examined using field‐emission scanning electron microscopy (FE‐SEM, HITACHI Ltd. S‐4800) at acceleration voltages of 5 and 10 kV, respectively. Energy dispersive X‐ray spectroscopy (EDS) was used to verify the film composition. Furthermore, X‐ray photoelectron spectroscopy (XPS, Thermo Fisher Scientific K‐Alpha) was performed for more detailed chemical composition analysis in the energy range of 0–1350 eV with a step size of 1 eV utilizing Al Kα source. X‐ray diffraction (XRD, Rigaku Corporation SmartLab) analysis was also performed to confirm the crystal structure of the deposited film in the angular range of 10–90° with a step size of 0.05° using a Cu target. Atomic force microscopy (AFM, Jupiter XR, Oxford Instruments) was operated in tapping mode for 5 × 5 µm^2^ scale.

### Electrochemical Measurements

To test the performance of TF‐SOFCs, two different stations were used for each cell size. A customized cell test station was employed to evaluate the electrochemical performance of the 1 × 1 cm^2^ anode‐supported SOFCs with n‐AFL. Dry hydrogen was fed to the anode at a flow rate of 150 sccm and the same amount of air was supplied to the cathode throughout the evaluation. A potentiostat (Bio‐logic SP‐300) was used to conduct linear sweep voltammetry (LSV) and electrochemical impedance spectroscopy (EIS) in the temperature range of 500–650 °C. LSV analysis was conducted from the open circuit voltage (OCV) to 0.4 V with a scan rate of 20 mV s^−1^. Electrochemical impedance spectroscopy (EIS) was performed at OCV, 0.8 V, and 0.6 V, controlling the frequency of the sinusoidal AC amplitude to 50 mV in the frequency range of 1 Hz–1 MHz. The large‐area cell was tested from 500–600 °C with 50 °C interval with EnergyLab XM potentionstat and booster (Solartron Analytical, Apps‐XM, Booster). Air and H_2_ (1.6 lpm) were supplied to the cathode and anode, respectively.

## Conflict of Interest

The authors declare no conflict of interest.

## Author Contributions

K.J., S.O., and J.H.L contributed equally to the study. H.J.K., H.K., S.E.J., and J.L. contributed to drafting the manuscript. B. C. Y., J. Y., and D. W. S. performed the experiments. W.P., J. W. S., Y. B. K., S.Y., and J. A. designed the experiments and analyzed the data. All authors contributed to the discussion and analysis of the results presented in the manuscript.

## Supporting information



Supporting Information

## Data Availability

The data that support the findings of this study are available in the supplementary material of this article.
