# Supplementary material for: Large Area High‐Performance Thin Film Solid Oxide Fuel Cell with Nanoscale Anode Functional Layer by Scalable Reactive Sputtering
Source: Adv Sci (Weinh). 2025 May 29;12(29):2502504. doi: 10.1002/advs.202502504 (PMC12362729; doi:10.1002/advs.202502504)
Supplement: Supplementary file 1 — Supporting Information [file ADVS-12-2502504-s001.docx]

Supplementary Information

**Large Area High-Performance Thin Film Solid Oxide Fuel Cell with Nanoscale Anode Functional Layer by Scalable Reactive Sputtering**

*Kyoungjae Ju^1,7^, Seongkook Oh^2,3,7^, Jong Hyuk Lee^4,7^, Hyong June Kim^1^, Hyunmin Kim^1^, Sung Eun Jo^1^, Juhwan Lee^1^, Byung Chan Yang^2^, Jisung Yoon^4^, Dong Won Shin^5^, Wanwoo Park^5^, Ji-Won Son^2,3,6^, Young-Beom Kim^4,*^, Sungeun Yang^2,3,*^, Jihwan An^1,*^*

**Affiliation**

*^1^ Department of Mechanical Engineering, Pohang University of Science and Technology (POSTECH), Republic of Korea.*

*^2^ Energy Material Research Center, Korea Institute of Science and Technology (KIST), Republic of Korea.*

*^3^ Nanomaterials Science and Engineering, Korea University of Science and Technology (UST), Republic of Korea.*

*^4^ Department of Mechanical Engineering, Hanyang University, Republic of Korea*

*^5^ Research and Development Team, AVACO Co., Ltd., Republic of Korea.*

*^6^ Graduate School of Energy and Environment (KU-KIST Green School), Korea University, Republic of Korea.*

*^7^ These authors contributed equally to this work.*

* Corresponding authors.
E-mail: ybkim@hanyang.ac.kr (Y.-B. K.), syang@kist.re.kr (S. Y.) and jihwanan@postech.ac.kr (J. A.)


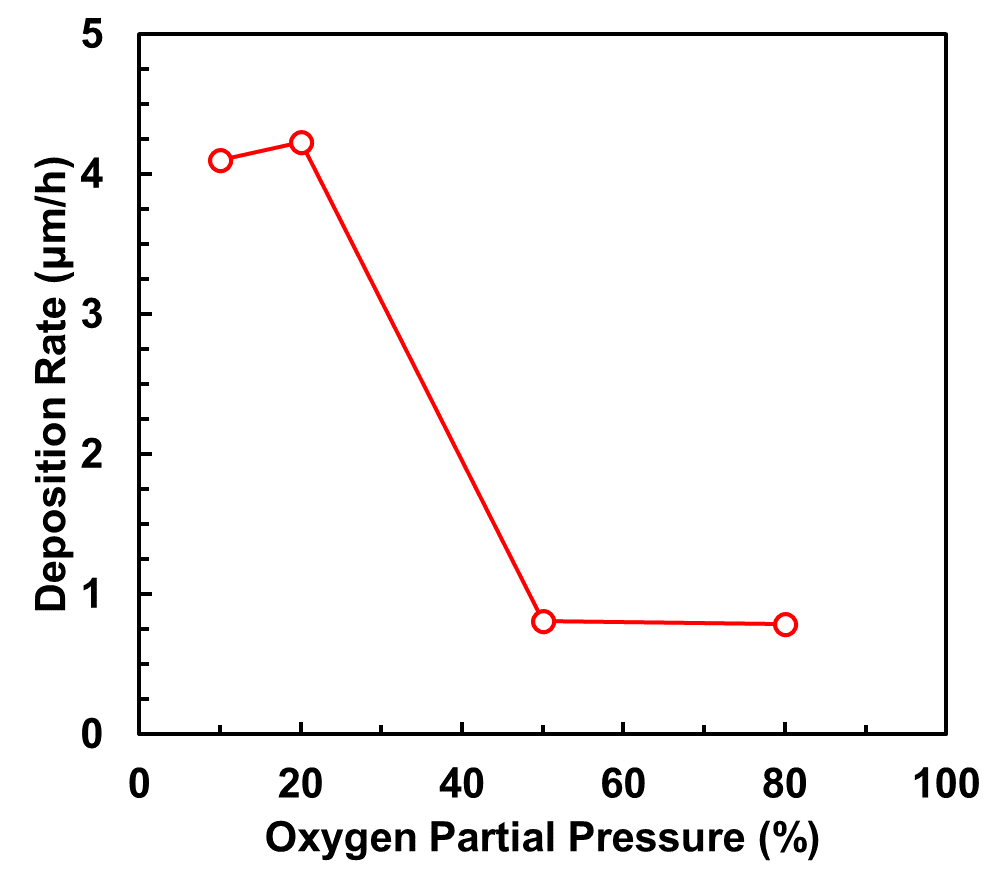


**Figure S1** Deposition rate depends on the oxygen partial pressure with constant sputtering power of 2 kW.


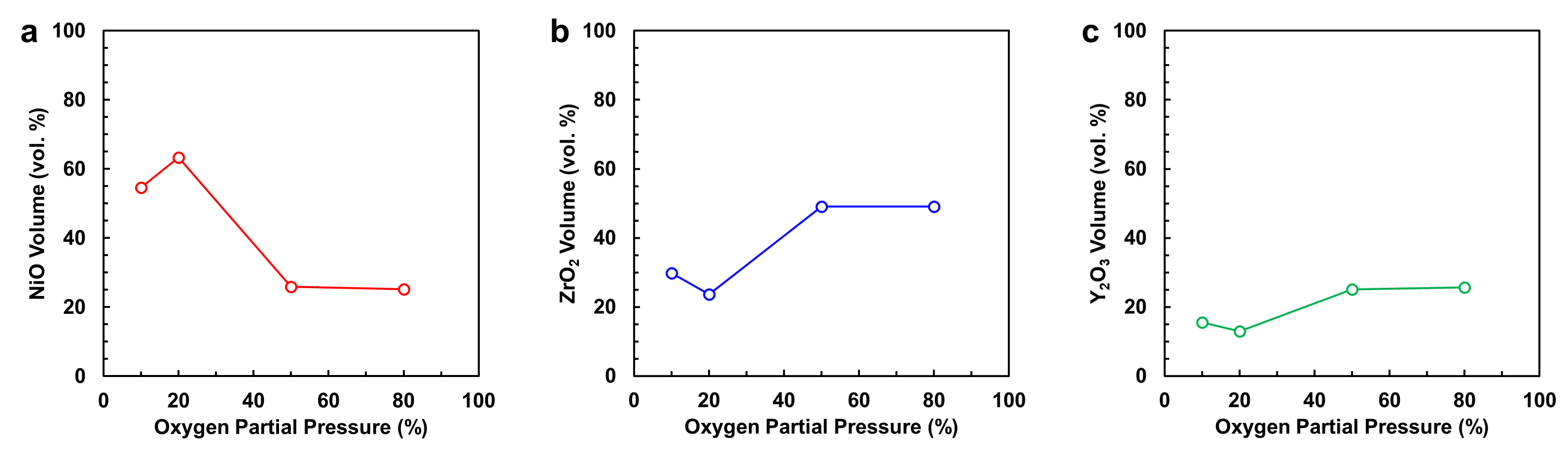


**Figure S2** Volumetric contents of 1200℃ annealed n-AFLs as a function of oxygen partial pressure. (estimated from XPS analysis)


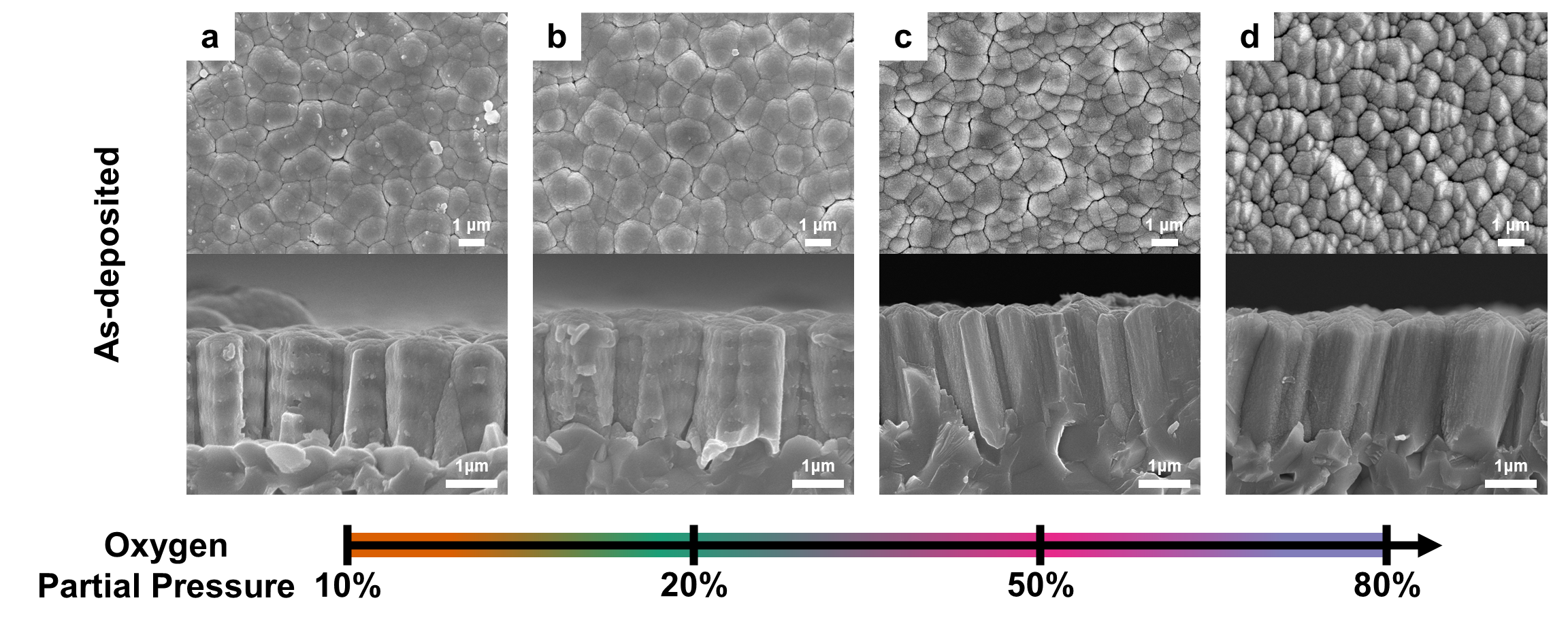


**Figure S3** SEM image of as-deposited NiO-YSZ n-AFL at different oxygen partial pressure conditions with constant sputtering power of 2 kW.

**
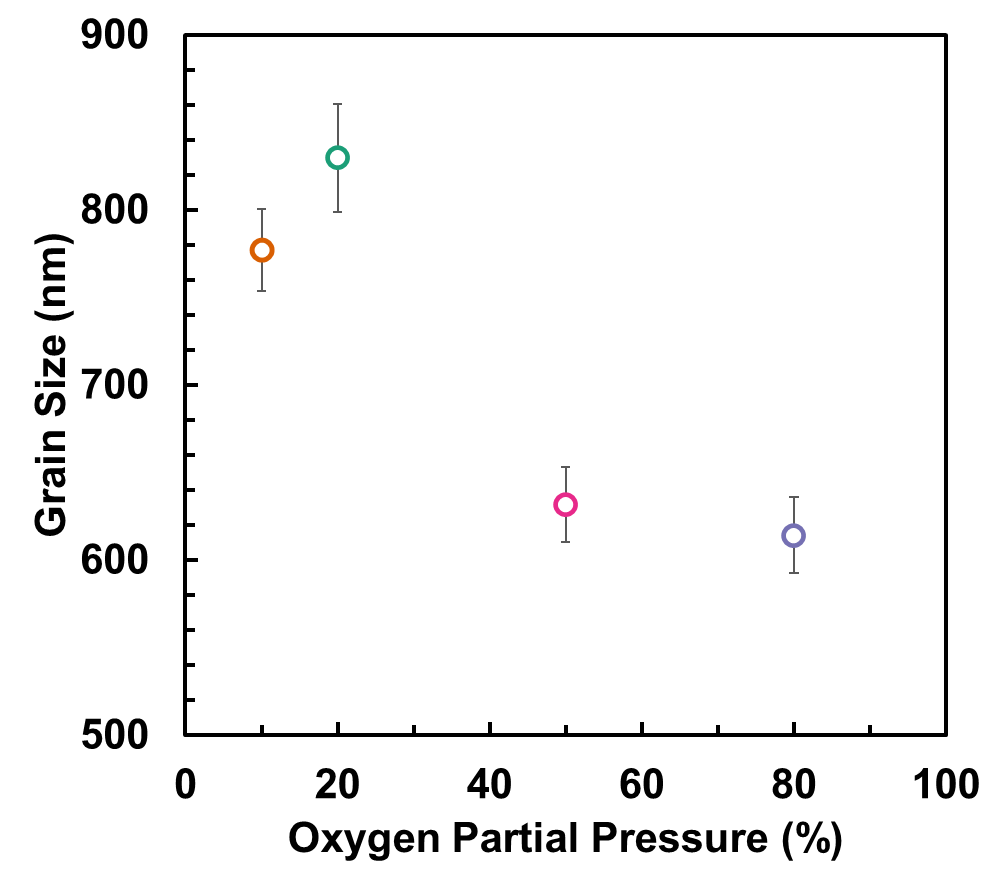
**

**Figure S4** Average grain size of as-deposited NiO-YSZ n-AFL at different P_O2_ conditions.

**
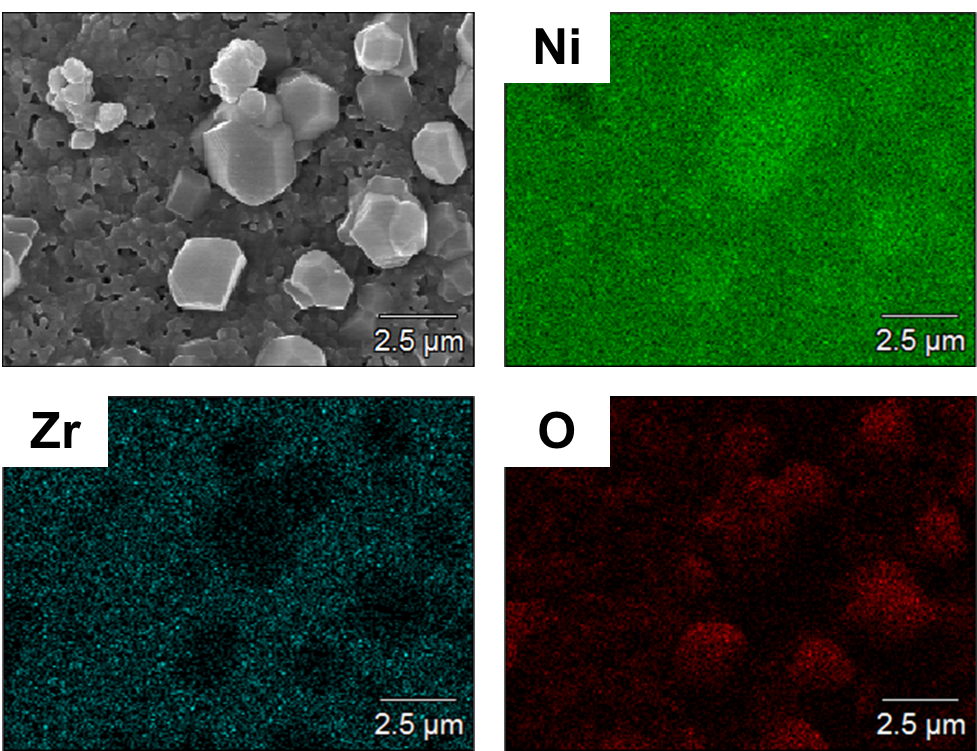
**

**Figure S5** SEM-EDS images of annealed n-AFL fabricated with 20% P_O2_.

**
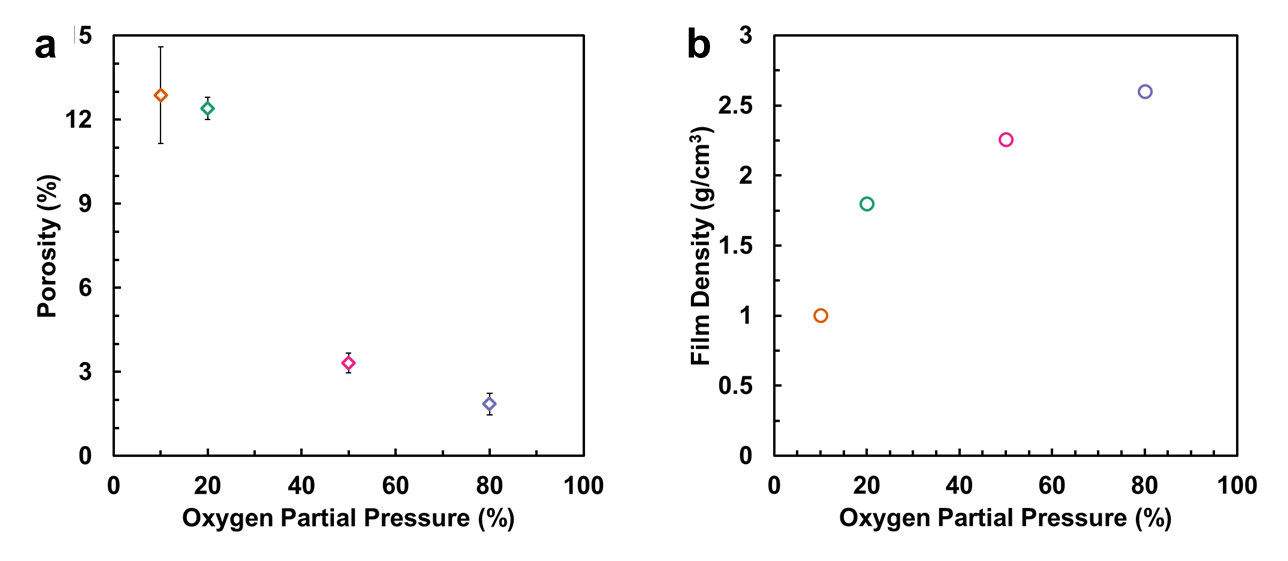
**

**Figure S6** (a) Porosity and (b) film density of annealed NiO-YSZ n-AFL at different P_O2_ conditions.

**
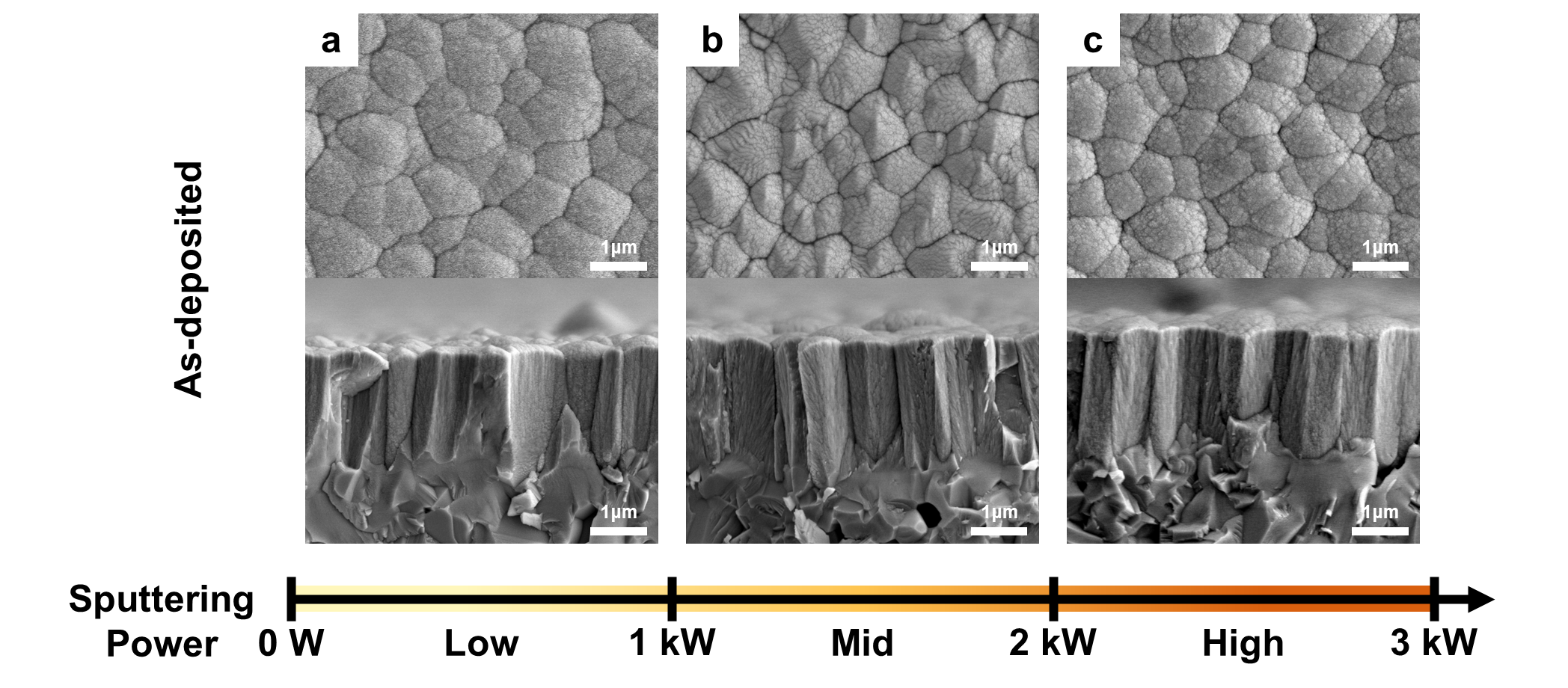
**

**Figure S7** SEM image of as-deposited NiO-YSZ n-AFL at different sputtering power conditions with constant oxygen partial pressure of 80%.

**
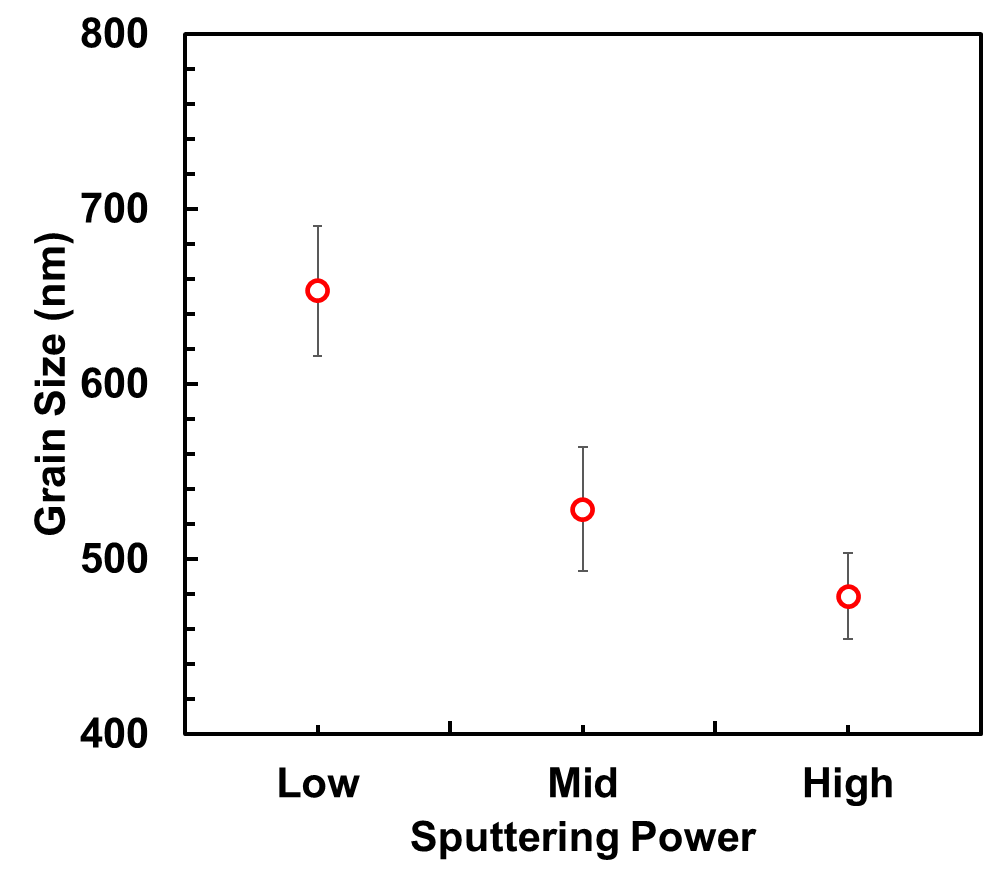
**

**Figure S8** Average grain size of as-deposited NiO-YSZ n-AFL at different sputtering power.

**
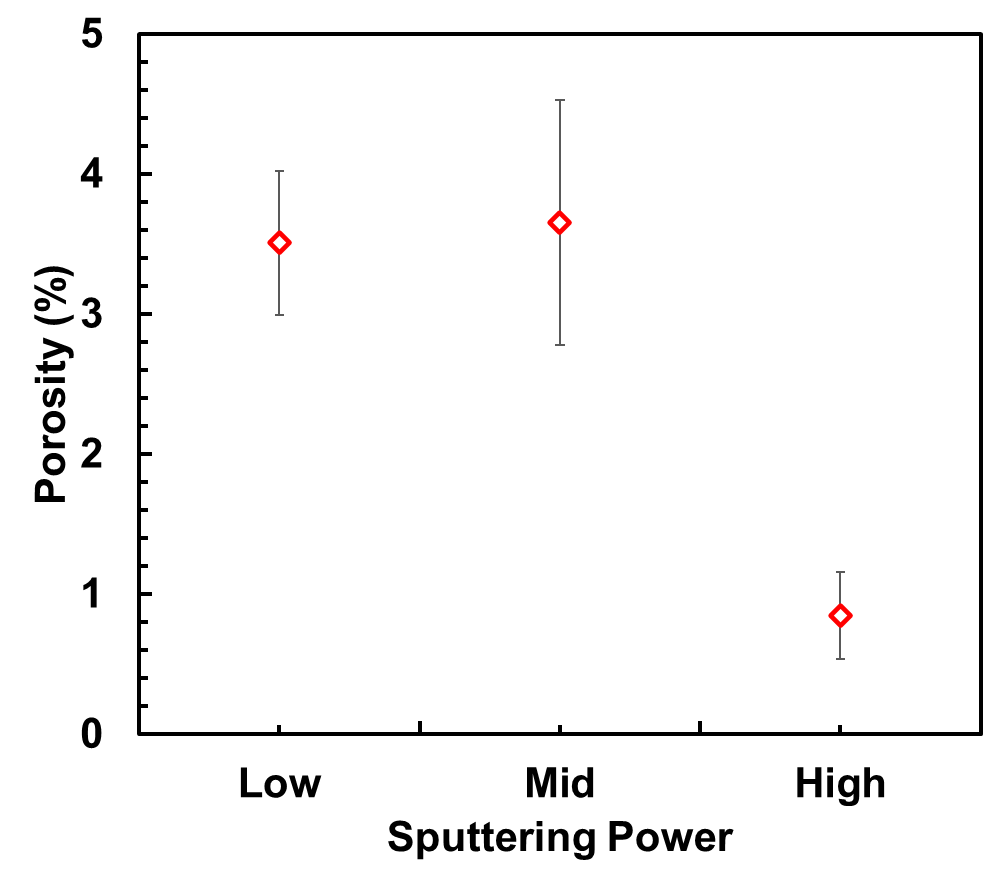
**

**Figure S9** Average grain size of as-deposited NiO-YSZ n-AFL at different sputtering power.


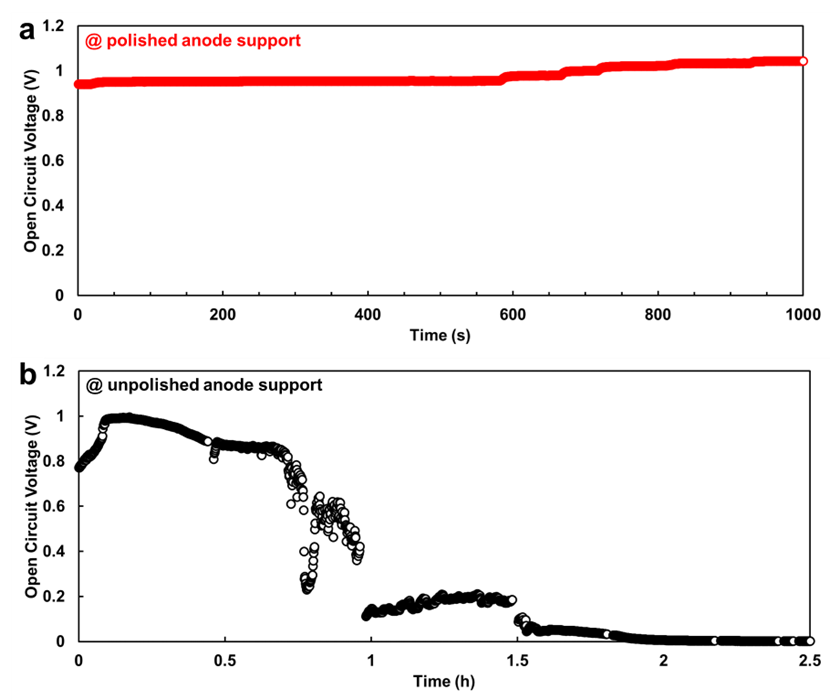


**Figure S10** Open circuit voltage trends after the pre-reduction of the TF-SOFCs without n-AFL using (a) polished and (b) unpolished anode supports.

**
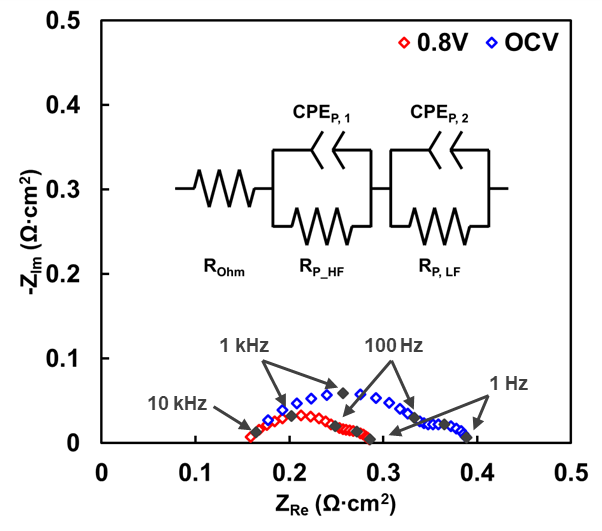
**

**Figure S11** Example of EIS spectra that shows the dependency on the cell voltage. (n-AFL implemented TF-SOFC, @ 600℃)

**
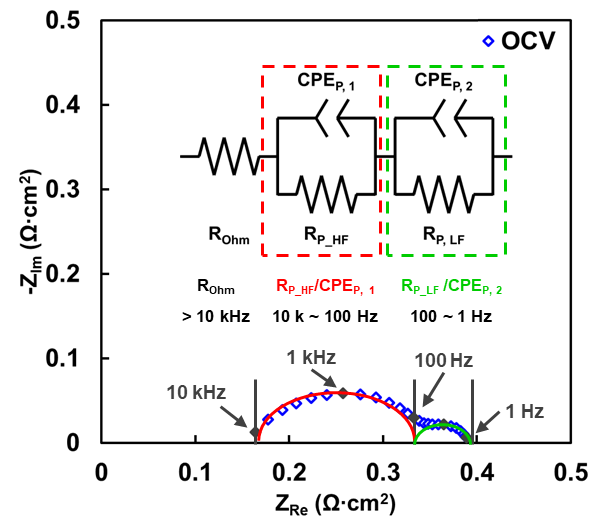
**

**Figure S12** Example of EIS spectra that can be deconvoluted into ohmic, high-, and low-frequency polarization impedances. (n-AFL implemented TF-SOFC, @ 600℃)

**
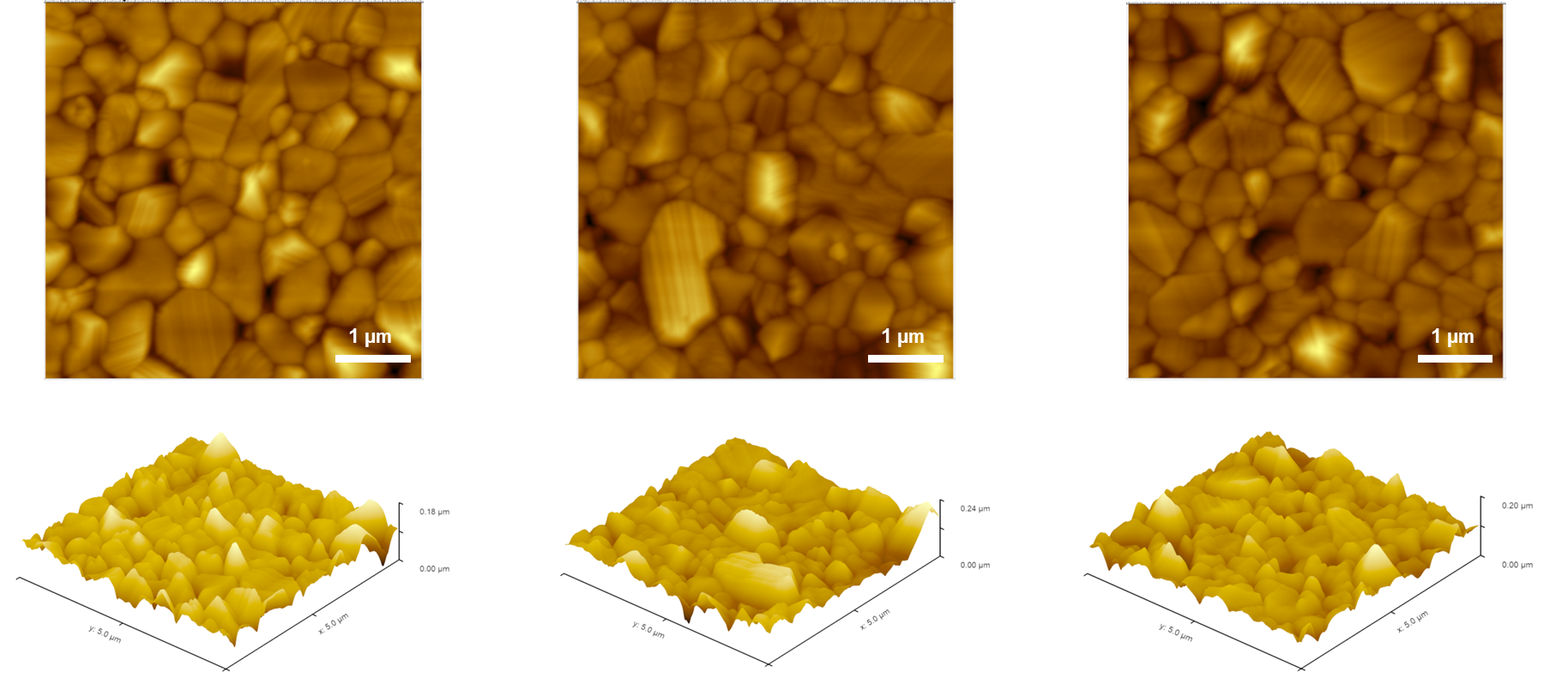
**

**Figure S13** AFM images and 3D morphologies of polished Ni-YSZ anode supports.

**
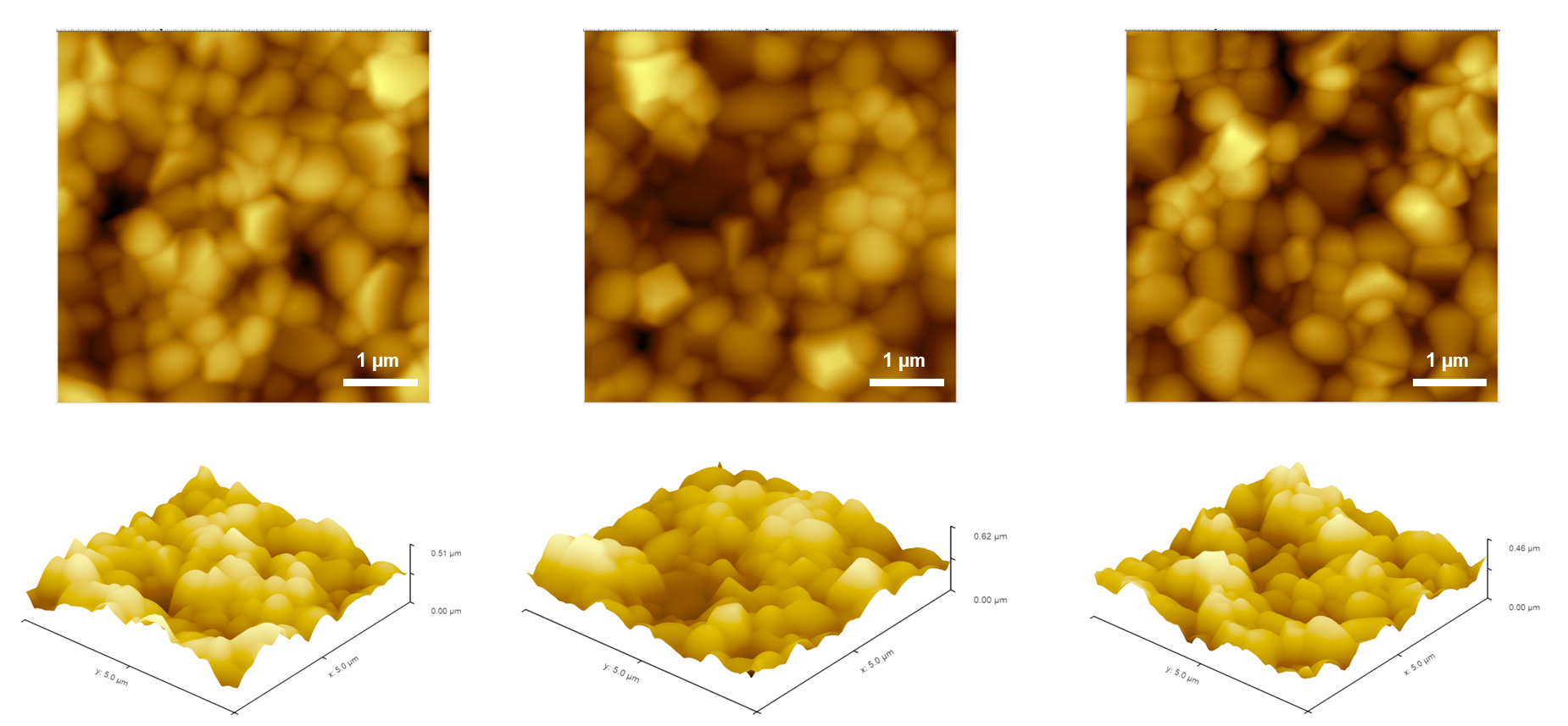
**

**Figure S14** AFM images and 3D morphologies of unpolished Ni-YSZ anode support.

**
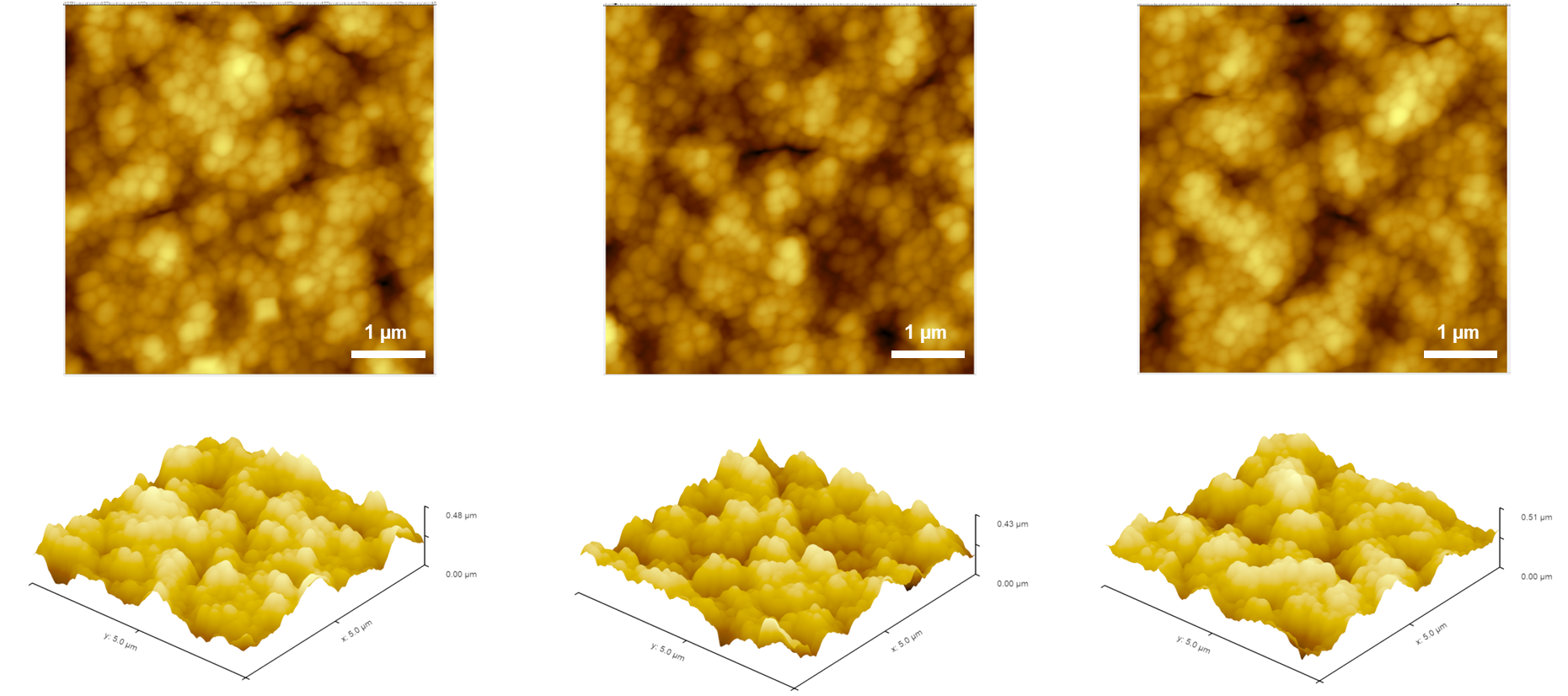
**

**Figure S15** AFM images and 3D morphologies of reactively sputtered n-AFL on the unpolished Ni-YSZ anode support.

**
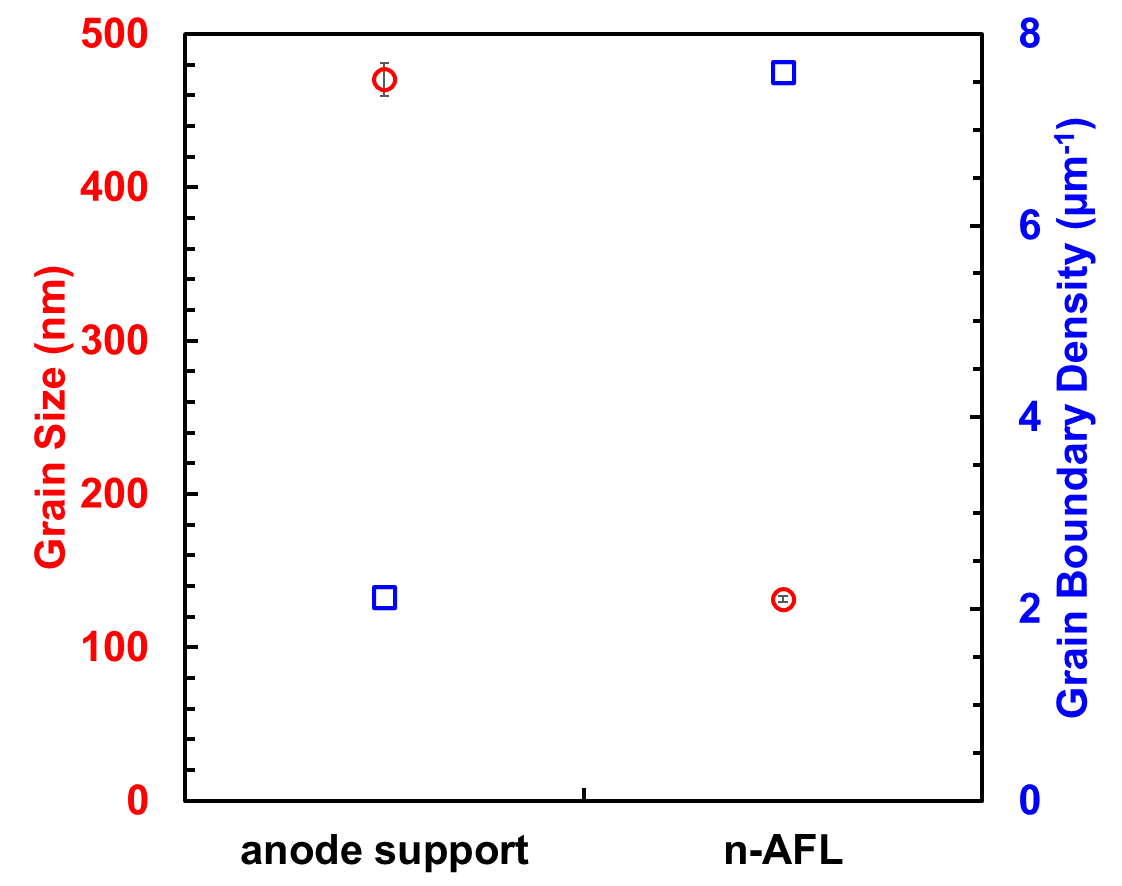
**

**Figure S16** Average diameter of grains on anode support and n-AFL surface, and corresponding grain boundary density.

**
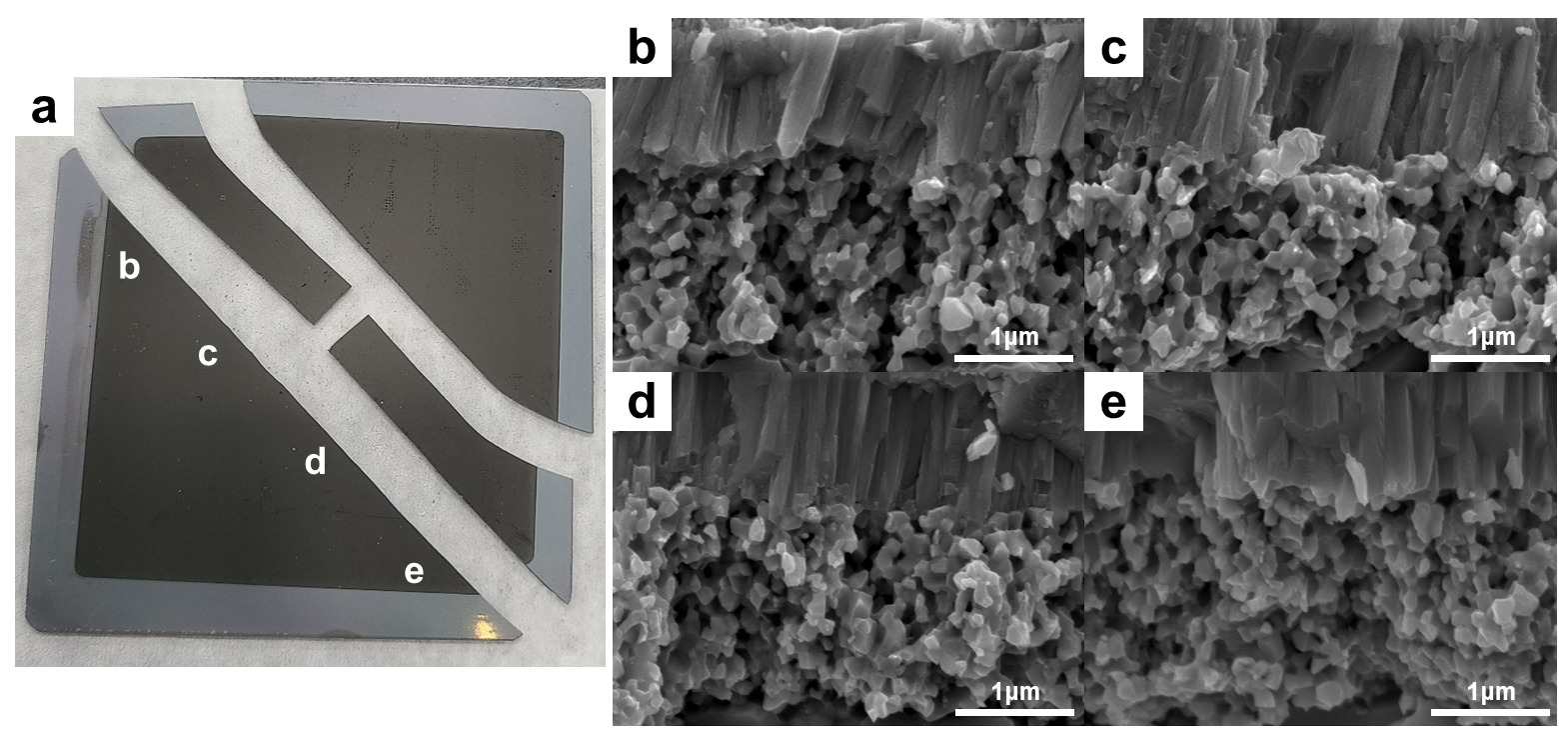
**

**Figure S17** (a) Digital image of the large-area cell after the cell test. (b-e) Cross-sectional SEM images from 4 different points along the diagonal of the large-area cell.

**
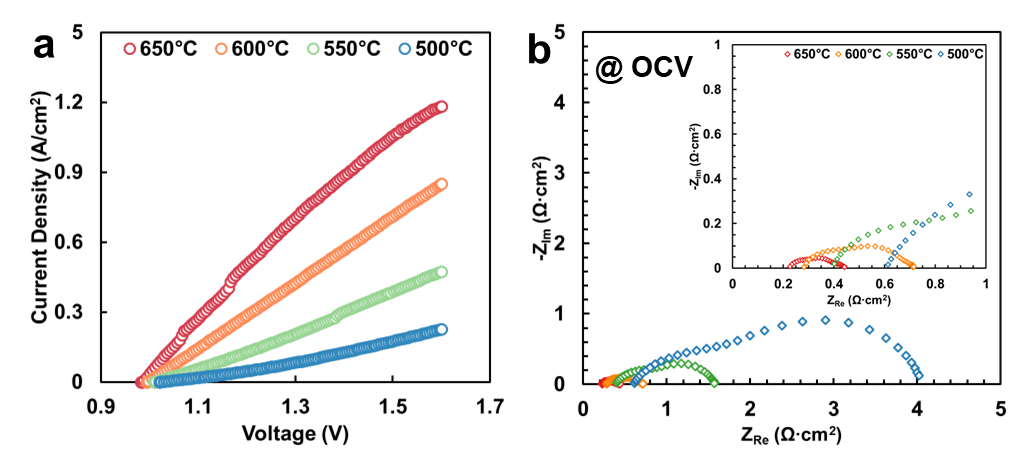
**

**Figure S18** (a) Polarization curves of the solid oxide electrolysis cell(SOEC) with the n-AFL and (b) corresponding EIS spectra. During the electrolysis test, 50 % humidified hydrogen was supplied to the fuel electrode where n-AFL was located, while 20 % oxygen was supplied to the air electrode.
